# Differentially Methylated Genes in Muscle Associated with Gait Speed in a Non-Human Primate Model of Aging

**DOI:** 10.1093/geroni/igab046.2504

**Published:** 2021-12-17

**Authors:** Ellen Quillen, Brett Frye, Bethany Wildeman, Maggie Stainback, Thomas Register, Carol Shively

**Affiliations:** Wake Forest School of Medicine, Winston Salem, North Carolina, United States

## Abstract

Age-related changes in DNA methylation are potent regulators of gene expression and may in part explain the onset of disease and disability. Vervet monkeys are a well-described model of neurocognitive and physical aging. Like humans, gait speed declines with age in vervets, and variability in gait speed in older animals is associated with age-related musculoskeletal and cognitive decline. To identify methylation patterns linked to aging-related physical decline, we investigated differentially methylated loci in vastus lateralis biopsies of 29 female vervets aged 8-28 years (~25-90 years in humans). We evaluated 107,490 loci on the Illumina Infinium Methylation EPIC Human Array that aligned with high fidelity to the vervet genome using the R package minfi and fit generalized linear mixed models to account for underlying genetic relatedness. We found 13 CpG methylation sites associated with 12 genes (CALCR, EBF4, GDNF, GMCL1, HAND1, HOXC10, IRX2, LBX2, MPPED2, SHISA6, SOX2, and WNT2) significantly differentially methylated with gait speed. Increased methylation was negatively associated with gait speed for all loci except GMCL1, reflecting the pattern of global hypermethylation of skeletal muscle tissue with age. Several of the associated genes are involved in development and differentiation including HOXC10 and LBX2, which regulates myoblast migration. CACNG8 regulates voltage-dependent calcium gated channels, and GDNF promotes motor neuron innervation of skeletal muscle. Most associations with muscle phenotypes are novel, but several have been linked to age-related bone diseases. We are currently evaluating the relationships of these differentially methylated loci with muscle mRNA expression and protein abundance.

